# QSAR Modeling of COX -2 Inhibitory Activity of Some Dihydropyridine and Hydroquinoline Derivatives Using Multiple Linear Regression (MLR) Method

**Published:** 2017

**Authors:** Somaye Akbari, Tannaz Zebardast, Afshin Zarghi, Zahra Hajimahdi

**Affiliations:** a *Department of Pharmaceutical Chemistry, Faculty of Pharmaceutical Chemistry, Pharmaceutical Sciences Branch, Islamic Azad University, Tehran, Iran (IAUPS)*; b *Department of Medicinal Chemistry, School of Pharmacy, Shahid Beheshti University of Medical Sciences, Tehran, Iran.*

**Keywords:** COX-2 inhibitors, 1, 4-Dihydropyridines, 5-Oxo-1, 4, 5, 6, 7, 8 hexahydroquinolines, Multiple linear regression, QSAR

## Abstract

COX-2 inhibitory activities of some 1,4-dihydropyridine and 5-oxo-1,4,5,6,7,8-hexahydroquinoline derivatives were modeled by quantitative structure–activity relationship (QSAR) using stepwise-multiple linear regression (SW-MLR) method. The built model was robust and predictive with correlation coefficient (R^2^) of 0.972 and 0.531 for training and test groups, respectively. The quality of the model was evaluated by leave-one-out (LOO) cross validation (LOO correlation coefficient (Q^2^) of 0.943) and Y-randomization. We also employed a leverage approach for the defining of applicability domain of model. Based on QSAR models results, COX-2 inhibitory activity of selected data set had correlation with BEHm6 (highest eigenvalue n. 6 of Burden matrix/weighted by atomic masses), Mor03u (signal 03/unweighted) and IVDE (Mean information content on the vertex degree equality) descriptors which derived from their structures.

## Introduction

Non-steroidal anti-inflammatory drugs (NSAIDs) are a crucial category of medicine that used for the treatment of pain, fever, inflammation and arthritis-associated disorders. The main therapeutic mechanism of NSAIDs action arise from inhibition of biosynthesis of prostaglandins (PGs) by cyclooxygenase enzyme (COX). COX enzyme catalyzes the first step of the biosynthesis of PGG2 from arachidonic acid to generate PGH2. In the next step of enzyme catalysis PGH2 is converted to other prostaglandins and thromboxanes that are involved in diverse biological responses (1, 2). On the basis of crystal structures, the two isoforms, COX-1 and COX-2 have been identified. Cyclooxygenase-1 (COX-1) mainly associated with prostaglandin production in gastric mucosa and is constitutive but COX-2 is upregulated in response to inflammatory stimuli and involved in pathologic processes (3, 4). All classical NSAIDs are capable of inhibiting both COX-1 and COX-2. As a result, these drugs are associated with side effects such as gastric ulceration, bleeding and renal dysfunction (5). Accordingly, introducing the selective COX-2 inhibitor provides therapeutic benefit in inflammation without gastric ulceration. Within the last decades, various structural classes of selective COX-2 inhibitors were reported in the literature (6-12). In addition to their anti-inflammatory activity, selective COX-2 inhibitors are considered as attractive molecules for anti-cancer research and neurological disorders such as Parkinson and Alzheimer’s diseases (13-15). Thus, design and discovery of new COX-2 inhibitors without toxic side effects is required. For these reasons, in this project, as part of our ongoing program on discovering novel selective COX-2 inhibitors, we selected some 1,4-dihydropyridine and 5-oxo-1,4,5,6,7,8-hexahydroquinoline derivatives which have been synthesized and evaluated as selective COX-2 inhibitors in our laboratory for quantitative structure–activity relationship (QSAR) study (16, 17). 

QSAR is an applicable approach in computer-aided drug design and development. The major role of QSAR study is correlating biological activities of chemicals to their structural features. Obtained validated QSAR models are utilized for quantitatively predicting the activities of candidate structures. Therefore, extra costs for drug design and discovery such as unnecessary synthesis processes and biological activity assays may be avoided (18-21).

In this study, we employed the stepwise (SW) selection method for the variable selection in the multiple linear regression (MLR) method to build an accurate quantitative relationship between the molecular structure and the COX-2 inhibitory activity of some 1,4-dihydropyridine and 5-oxo-1,4,5,6,7,8-hexahydroquinoline derivatives. The findings can provide helpful information for designing new biologically active molecules.

## Experimental

*Data set*

Two series of potent 1, 4-dihydropyridine and5-oxo-1,4,5,6,7,8-hexahydroquinoline derivatives (21 compounds) which have been synthesized and evaluated as selective COX-2 inhibitors in our laboratory was taken for the study (16, 17). All the biological data expressed as IC_50 _were converted into pIC_50_ (-log IC_50_) values. The total set of molecules was randomly separated into a training set (17 compounds) for generating QSAR model and a test set (4 compounds) for validating the quality of the model. The general chemical structures and biological activity values of all of the compounds are shown in Table 1.

*Molecular descriptors and geometry optimization*

The 2D chemical structures of the molecules were built using the HyperChem 8.0 software (version 8.0; HyperChem, Alberta, Canada). The pre-optimization was conducted using the molecular mechanics force field (MM+) procedure included in HyperChem, and then semi-empirical method AM1 using the Polak-Ribiere algorithm until the root mean square gradientwas 0.001 Kcal.mol^-1^ was applied to optimize the molecules geometry. DRAGON software was used to calculate the descriptors and finally 1497 molecular descriptors such as constitutionaldescriptors, topological descriptors, molecular walk counts, BCUT descriptors, Galves topological charge indices, 2D autocorrelations, charge descriptors, aromaticity indices, Randic molecular profiles, geometrical descriptors, 3D-MoRSE descriptors, WHIM descriptors, GETAWAY descriptors, empirical descriptors was extracted (22). 

The calculated descriptors were first analyzed for the existence of constant or near constant variables. The detected ones were then removed. Secondly, the descriptors correlation with each other and with the activity (pIC_50_) of the molecules was examined and the collinear descriptors (*i.e.* correlation coefficient between descriptors is greater than 0.9) were detected. Among the collinear descriptors, the one exhibiting the highest correlation with the activity was retained and others were removed from the data matrix and finally 366 descriptors were remained.

## Results

**Figure 1:**
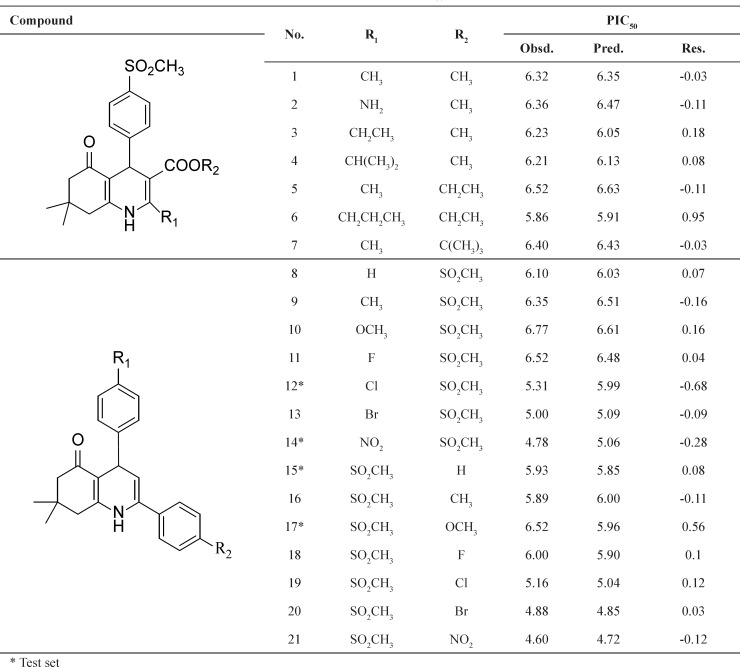
Influences of the number of descriptors on the R2 and SEE of the regression model

For the selection of the most important descriptors, stepwise method-based MLR was used. According to rule of thumb, at least five compounds should be included in the equation for every descriptor. To investigate the optimum number of descriptors to be used in the equation, a graph between numbers of descriptors against statistical parameters (R^2^ and Standard Error of Estimate (SEE)) was plotted (Figure 1). Figure 1 shows that R^2^ increased with the increasing number of descriptors. However, the values of SEE decreased with the increasing number of descriptors. As can be seen, R^2^ and SEE remain almost parallel to the number of descriptors after three parameters and higher order models. This shows that the most suitable models are three parametric models.

The MLR analysis with a stepwise selection was carried out to relate the pIC_50_ to a three set of descriptors. The SPSS software (version 21.0; SPSS Inc., Chicago, IL, USA) was employed for the MLR analysis). It is described by the following equation:

*pIC*_50_ *= 9.370 (± 3.76) – 7.397 (± 0.407) BEHm6 – 0.208 (± 0.19) Mor03u + 8.794 (± 1.685) IVDE*

The built model produced good results for the training set and the test set (Tables 1 and 2).

It can be seen that the SW-MLR equation has acceptable quality and can predict the activity of train and test set with R^2 ^= 0.972 and R^2^ = 0.531, respectively. The plots of the predicted pIC_50 _versus the experimental pIC_50_, obtained by the SW-MLR modeling, are demonstrated in 

Figure 2.

The cross-validation analysis was performed using leave-one-out (LOO) method in which one compound is removed from the data set and the activity is correlated using the rest of the data set. The cross-validated R^2^ (Q^2^) was found to be very close to the value of R^2^ for the train set and hence these models can be termed as statistically significant. The obtained statistical parameter of the leave-one-out cross-validation test (Q^2^) on SW-MLR model was 0.943 and RMSE of cross-validation was 0.13, which indicates reliability of the proposed model. 

The selected variables of SW-MLR model are shown in Table 3.

**Figure 2:**
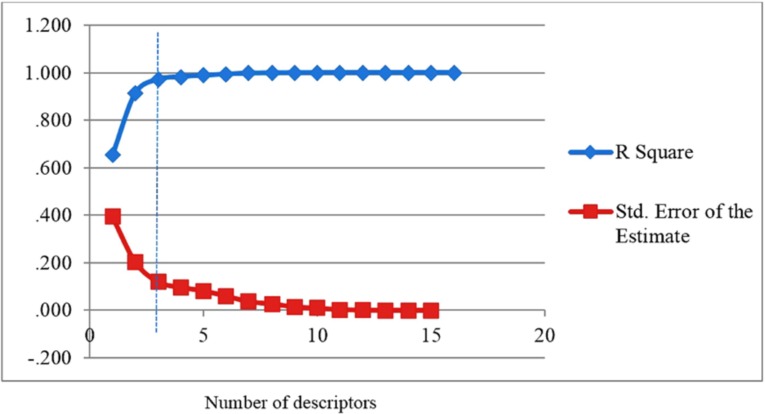
The predicted pIC50 values by the SW-MLR modeling versus the experimental pIC50 values.

**Figure 3:**
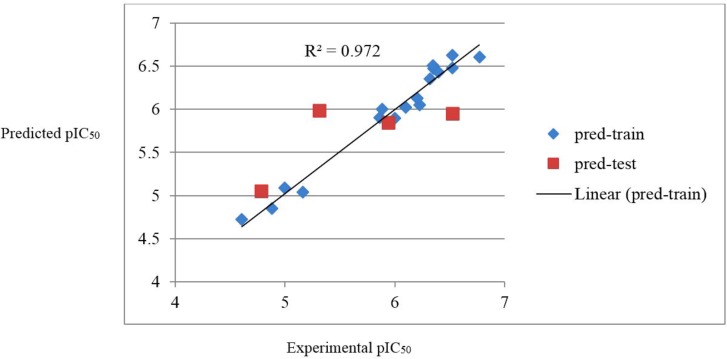
The William plot for the SW-MLR model

**Figure 4:**
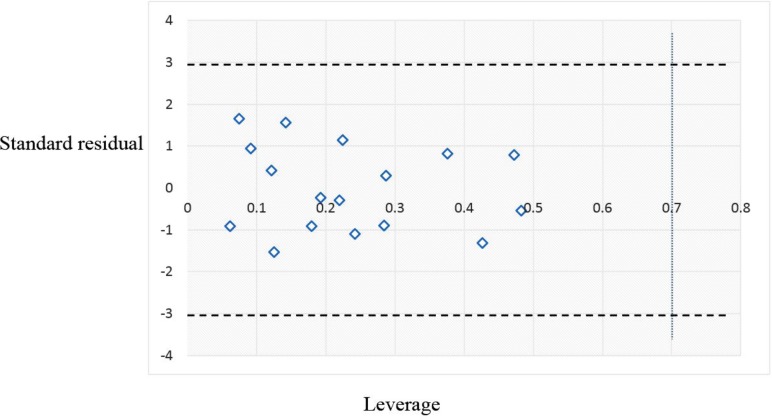
Standardized coefficients versus descriptor values in MLR

**Table 1:** Chemical structures and the corresponding observed and predicted pIC_50_ values by SW-MLR method.

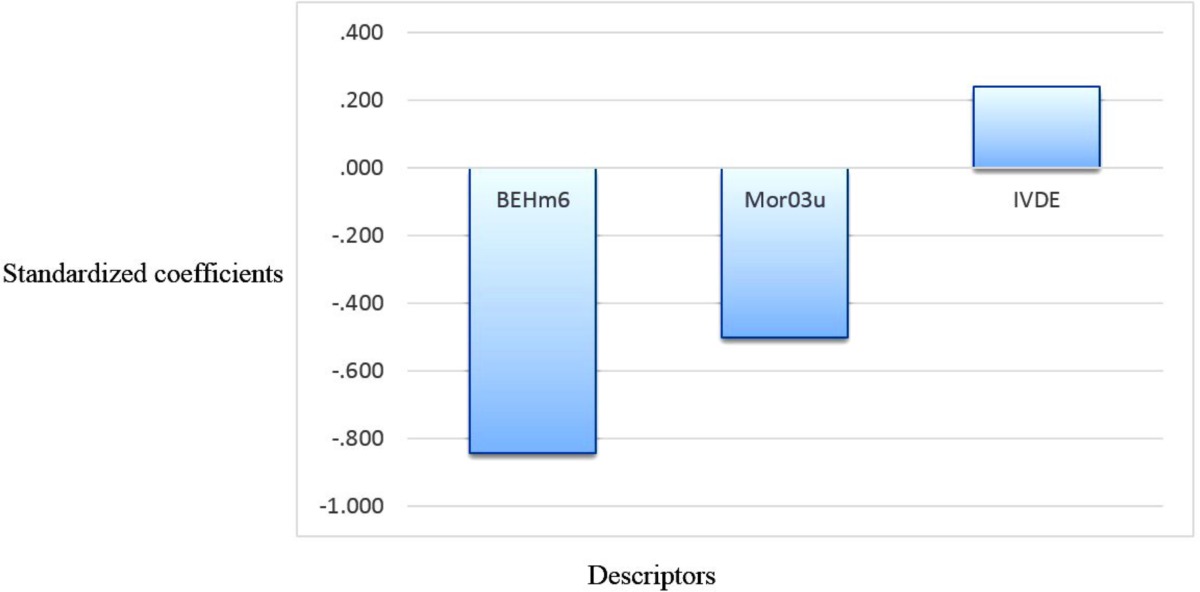

*Test set

**Table 2:** Statistical parameters of SW-MLR model

**Training Set**		**Test Set**	**F (3, 13)**	**Q** ^2^_LOO_	**RMSE Q** ^2^_LOO_
**SEE**	**R** ^2^	**R** ^2^	151.3	0.94	0.13
0.12	0.97	0.531			

**Table 3:** The descriptor values were used in model construction

**Name**	**BEHm6**	**Mor03u**	**IVDE**
1	3.085	-6.333	2.102
2	3.07	-6.379	2.102
3	3.167	-8.315	2.09
4	3.202	-8.583	2.122
5	3.128	-9.681	2.09
6	3.215	-10.729	2.057
7	3.164	-9.581	2.1
8	3.083	-7.199	2.043
9	3.083	-7.83	2.083
10	3.083	-8.38	2.081
11	3.083	-7.66	2.083
12*	3.174	-8.569	2.083
13	3.294	-8.497	2.083
14*	3.167	-4.367	2.07
15*	3.115	-7.492	2.043
16	3.146	-7.588	2.083
17*	3.155	-7.806	2.081
18	3.133	-6.644	2.083
19	3.221	-5.683	2.083
20	3.295	-7.389	2.083
21	3.213	-4.398	2.07

* Test Set

**Table 4:** Correlation coefficient matrix of the selected descriptors by SW-MLR

	**BEHm6**	**Mor03u**	**IVDE**
BEHm6	1.00	-0.06	0.05
Mor03u		1.00	-0.11
IVDE			1.00

**Table 5:** R^2^ and Q^2^_LOO_ values of SW-MLR after several Y-randomization test

**Iteration**	**SW-MLR**	
	**R** ^2^	**Q** ^2^_LOO_
1	0.49	-0.08
2	0.09	-0.96
3	0.31	-0.39
4	0.08	-0.76
5	0.15	-0.49
6	0.08	-0.52
7	0.05	-0.96
8	0.12	-0.70
9	0.09	-0.76
10	0.42	0.10

**Table 6:** Details of name of the descriptors were used in model construction

**Descriptor name**	**Explanation**	**Descriptor family**
BEHm6	highest eigenvalue n. 6 of Burden matrix/weighted by atomic masses	Molecular descriptors
Mor03u	Signal 03/unweighted	3D MORSE descriptors
IVDE	Mean information content on the vertex degree equality	Information indices

Collinearity is a major disadvantage in MLR analysis methods. Thus, the inter-correlation between the three selected descriptors in SW-MLR models was calculated. Results from Table 4 indicated that the absolute correlation coefficient value of each pair descriptors was less than 0.11. Therefore, selected descriptors by stepwise method were completely independent.

The robustness of the resulting model was further validated by applying Y-randomization test. Several random shuffles were performed on dependent variable (pIC_50_) and new QSAR model was built. The low R^2^ and Q^2^_LOO_ values show that the good results in obtained model regarded as reasonable and was not because of a chance correlation (Table 5).

After internal and external validation of the model, it cannot be claimed that this QSAR model is reliable for screening new compounds unless its domain of application is determined. The leverage along with the Williams plot is usually used to define applicability domain of a model. The Williams plot defines as the plot of the standardized residuals versus the leverage (*h*). In this plot, two horizontal lines and one vertical line mark a safety area. Compounds with standard residuals > 3 standard deviation units and leverage higher than the warning h^*^ are regarded as outliers. The leverage (*h*_i_) of every compound is calculated by following equation:

*h*_i_ *= x*_i_ *(X* ^T^ *X)* ^-1^ *x*_i_^T^

In this equation, *x*_i_ is the descriptor-row vector of the query molecule and *X* is the *k × n* matrix containing the *k* descriptor values for each one of the *n* training molecules. The critical leverage h* (the vertical line) is fixed at *3(k + 1)/n* (23, 24). From the Williams plot (Figure 3), it is obvious that all data points fall within the safety zone in both models. In addition, all compounds have the leverage lower than the warning h* value of 0.70. As a result, it can be said that the model is acceptable for prediction purpose.

## Discussion

QSAR results can provide useful chemical visions for designing new compounds. For this purpose, interpretation of the descriptors appeared in the resulting models was discussed below. The interpretation of the descriptors that appeared in SW-MLR model was extracted from Handbook of Molecular Descriptors (25-27). The chemical meaning of selected descriptors is also displayed in Table 6.

The relative significance of the descriptors presented in the QSAR model was determined based on their standardized regression coefficients. The calculated MLR coefficients cannot be used because the descriptors in final MLR model have not the same units. Standardized regression coefficients of selected descriptors in SW-MLR model are shown graphically in Figure 4.

BEHm6 (highest eigenvalue n. 6 of Burden matrix/weighted by atomic masses) is one of the BCUT descriptors which has appeared in the SW-MLR model. BCUT is the eigenvalue based descriptor noted for its utility in chemical diversity. The descriptor is based on a weighted version of the Burden matrix which takes into account both the connectivity as well as atomic properties of a molecule. The weights are a variety of atom properties placed along the diagonal of the Burden matrix. This descriptor displays a main negative sign, which indicates that the pIC_50_ is directly related to molecules atomic masses. Therefore, increasing the BEHm6 descriptor leads to decrease in its pIC_50_ value.

Mor03u is one of 3-D MoRSE discriptors that defines as molecular descriptors calculated by summing atom weights viewed by a different angular scattering function. These descriptors are based on the idea of obtaining information from the 3D atomic coordinates by the transform used in electron diffraction studies for preparing theoretical scattering curves. This descriptor has a negative effect on the inhibitory activity of analogs.

IVDE is one of information indices that defines as molecular descriptors calculated as information content of molecules, based on the calculation of equivalence classes from the molecular graph. Since it presented a positive sign in MLR equation, increasing in value of this descriptor will cause to increase of the activity (pIC_50_).

Summarizing, it is concluded that atomic masses, mean information content on the vertex degree equality and molecules shapes had important effects on the pIC_50_ of the compounds studied.

## Conclusion

In this study, SW-MLR was used to develop linear QSAR model for prediction of COX-2 inhibitory activity of 4-dihydropyridine (DHP) and 5-oxo-1,4,5,6,7,8-hexahydroquinoline derivatives. The built model displayed good correlations between the structure and activity of the studied compounds. The model was validated using LOO cross-validation, Y-randomization and external test set. The built model had a good self- and external-predictive power. Calculated applicability domain of model showed that obtained model was acceptable for prediction purpose. Based on QSAR models results, BEHm6 (highest eigenvalue n. 6 of Burden matrix/weighted by atomic masses), Mor03u (signal 03/unweighted) and IVDE (mean information content on the vertex degree equality) were found to be important factors controlling the COX-2 inhibitory activity.
